# An in vivo multimodal feasibility study in a rat brain tumour model using flexible multinuclear MR and PET systems

**DOI:** 10.1186/s40658-020-00319-6

**Published:** 2020-07-29

**Authors:** Chang-Hoon Choi, Carina Stegmayr, Aliaksandra Shymanskaya, Wieland A. Worthoff, Nuno A. da Silva, Jörg Felder, Karl-Josef Langen, N. Jon Shah

**Affiliations:** 1 Institute of Neuroscience and Medicine-4, INM-4, Forschungszentrum Jülich, Germany; 2 Institute of Neuroscience and Medicine-11, INM-11, JARA, Forschungszentrum Jülich, Germany; 3 Department of Nuclear Medicine, RWTH Aachen University Hospital, Aachen, Germany; 4 Jülich-Aachen Research Alliance (JARA)–Section JARA-BRAIN, Aachen, Germany; 5 JARA-BRAIN-Translational Medicine, Aachen, Germany; 6 Department of Neurology, RWTH Aachen University, Aachen, Germany

**Keywords:** MR PET, PET/MRI, MRI, FET PET, Multinuclear, Multimodal, Small animal

## Abstract

**Background:**

In addition to the structural information afforded by ^1^H MRI, the use of X-nuclei, such as sodium-23 (^23^Na) or phosphorus-31 (^31^P), offers important complementary information concerning physiological and biochemical parameters. By then combining this technique with PET, which provides valuable insight into a wide range of metabolic and molecular processes by using of a variety of radioactive tracers, the scope of medical imaging and diagnostics can be significantly increased. While the use of multimodal imaging is undoubtedly advantageous, identifying the optimal combination of these parameters to diagnose a specific dysfunction is very important and is advanced by the use of sophisticated imaging techniques in specific animal models.

**Methods:**

In this pilot study, rats with intracerebral 9L gliosarcomas were used to explore a combination of sequential multinuclear MRI using a sophisticated switchable coil set in a small animal 9.4 T MRI scanner and, subsequently, a small animal PET with the tumour tracer O-(2-[^18^F]-fluoroethyl)-L-tyrosine ([^18^F]FET). This made it possible for in vivo multinuclear MR-PET experiments to be conducted without compromising the performance of either multinuclear MR or PET.

**Results:**

High-quality in vivo images and spectra including high-resolution ^1^H imaging, ^23^Na-weighted imaging, detection of ^31^P metabolites and [^18^F]FET uptake were obtained, allowing the characterisation of tumour tissues in comparison to a healthy brain. It has been reported in the literature that these parameters are useful in the identification of the genetic profile of gliomas, particularly concerning the mutation of the isocitrate hydrogenase gene, which is highly relevant for treatment strategy.

**Conclusions:**

The combination of multinuclear MR and PET in, for example, brain tumour models with specific genetic mutations will enable the physiological background of signal alterations to be explored and the identification of the optimal combination of imaging parameters for the non-invasive characterisation of the molecular profile of tumours.

## Introduction

Proton magnetic resonance imaging (MRI) and positron emission tomography (PET) are well-established medical imaging modalities used in current routine clinical practice. MRI is typically employed to generate structural/anatomical images with both outstanding soft-tissue contrast and functional information. Alongside other MR techniques, the use of X-nuclei (nuclei other than ^1^H), such as sodium-23 (^23^Na) or phosphorus-31 (^31^P), which play a pivotal role in biochemistry and physiology, may provide complementary information with regard to cell membrane integrity and energy metabolism [1–3].

Interest in X-nuclei MRI has increased with recent technological developments [4–9] relating to a combination of increasing magnetic field strengths and advancements in MR coils and sequences. The sequences allow, for example, for the simultaneous acquisition of ^23^Na images weighted by restricted and non-restricted sodium concentration [10], or for ^1^H observed ^31^P editing [11] or saturation transfer of ^31^P metabolite signals [12]. These offer the opportunity to perform multinuclear MR imaging and spectroscopy with improved sensitivity, despite the challenges of detecting X-nuclei MR signals due to their relatively low natural abundance compared to the proton. MR-detectable ^23^Na signals can originate from both intra- and extracellular compartments of tissue. Any irregularity in tissue metabolism leads to remarkable ^23^Na signal intensity changes, and in tumours, the sodium concentration level is usually elevated [3, 13, 14]. ^31^P MR spectroscopy (MRS) provides information about a number of metabolites, such as adenosine triphosphate (ATP), phosphocreatine (PCr), phosphomonoester (PME), phosphodiester (PDE) and inorganic phosphate (Pi), with a relatively large chemical shift range (~ 30 ppm). Alterations in these metabolite peaks, either in intensity or in chemical shift, are also strongly related to a variety of pathological and neurodegenerative conditions [15–17].

PET uses a plethora of radioactive tracers to provide insight into molecular and metabolic processes with both a high level of specificity and sensitivity, whereby the latter can be, under optimal circumstances, a factor of 10^6^ greater than in MRI*.* For example, PET with radiolabelled amino acids, such as O-(2-[^18^F]-fluoroethyl)-L-tyrosine ([^18^F]FET) can provide diagnostic information in patients with gliomas [18–22]. [^18^F]FET shows low uptake in inflammatory lesions and healthy brain tissues but high uptake in gliomas due to an upregulation of specific amino acid transporters [22, 23]. This promises high specificity and high accuracy in the detection and differentiation of tumour cells and can be further improved when combined with MRI [24–26].

The optimal way to achieve maximum diagnostic accuracy would be to ascertain the best combination for non-invasive characterisation of unknown lesions by performing all possible imaging methods on a large number of patients. However, this is limited by the fact that patients are often seriously ill and can only tolerate a limited number of examinations. Therefore, sophisticated methods for experimental and translational imaging in animal models are of great importance for the provision of a direct comparison to the results of histological and physiological parameters. Due to recent technical advancements in genetic engineering, tumour models with a specific genetic profile can now be generated which allow imaging methods to be tested for the non-invasive prediction of the corresponding genetic profile [27]. In this context, we have developed a stand-alone, simple multinuclear MR system for imaging animals at ultra-high field [28] which is also capable of sequential acquisition with PET. In this proof of concept study, we explored the in vivo feasibility of multinuclear and multimodal experiments in an ultra-high field MR-PET set-up in rats using intracerebral 9L gliosarcomas. To our knowledge, this combination of multinuclear MR (using more than three nuclei) and PET for in vivo applications has never been demonstrated.

## Material and methods

### Multinuclear MRI and PET system

**Fig. 1:**
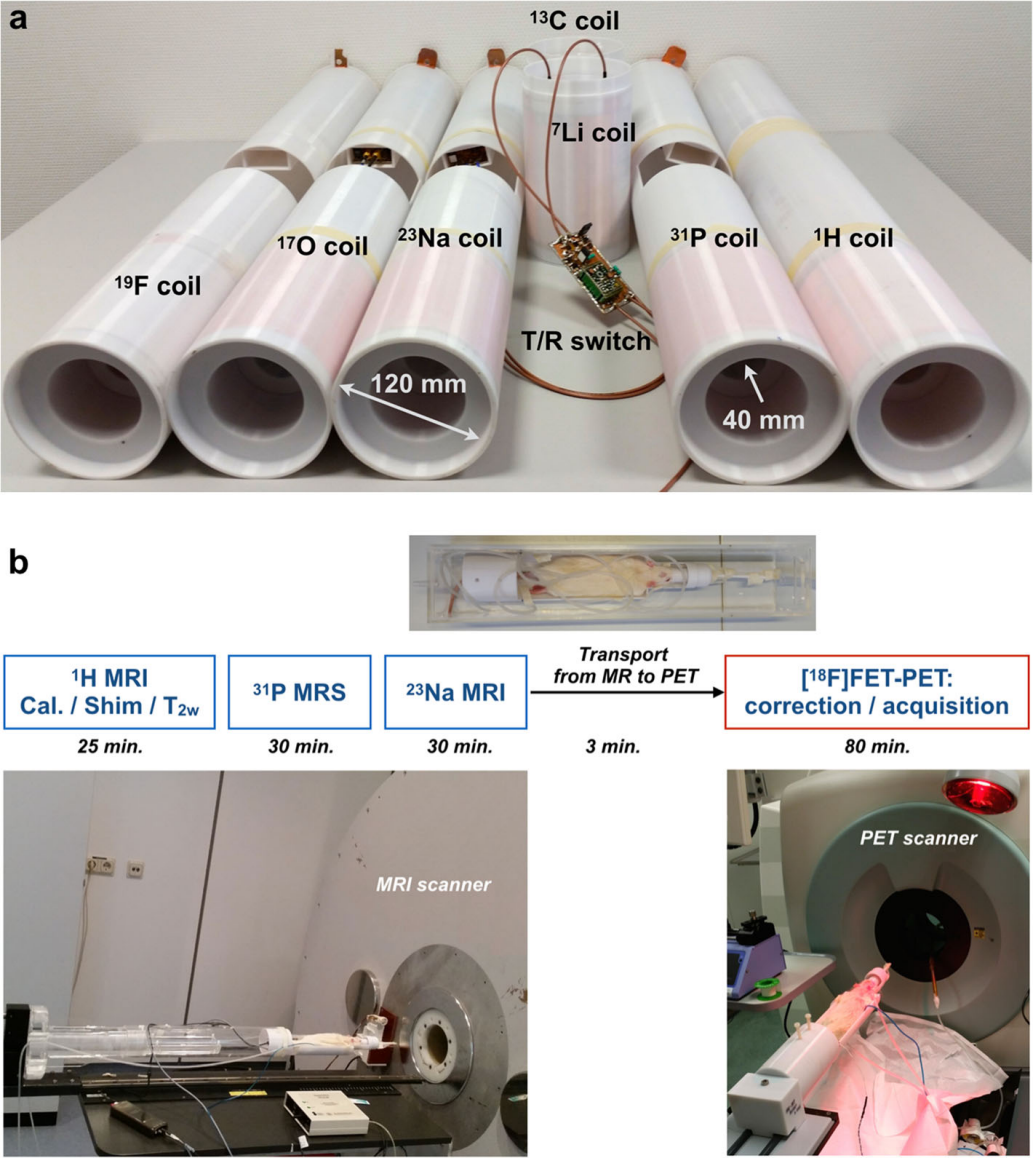
Picture of multinuclear MRI system showing RF coils and T/R switches (**a**), and MR, PET and transport systems with an example of measurement acquisition schemes (**b**)

All MR experiments were carried out on a home-integrated small animal dedicated 9.4 T MRI scanner. This system operates on clinical software from Siemens SYNGO [29]. Multinuclear MR acquisitions of ^1^H (399.7 MHz, corresponding Larmor frequency of ^1^H at 9.4 T), ^23^Na (105.8 MHz) and ^31^P (161.9 MHz) were achieved using the exchangeable multinuclear coil set (Fig. 1a), including a correspondingly tuned, quadrature birdcage head coil, a transmit/receive (T/R) switch and a pre-amplifier [28]. The benefit of this switchable multinuclear coil is that the maximum achievable coil sensitivity is attainable for any nuclei, which is hardly feasible using a conventional double- or triple-tuned coil configuration. In terms of the double-tuned coil, a reduction in sensitivity of approximately 25% is anticipated at both the 1H and X-nuclei frequencies due to the insertion of lossy decoupling units, such as LC traps or PIN-diodes. The performance of the coil only on a test phantom for the individual nucleus has been reported elsewhere [28]. The anaesthetised animal was positioned from the front side of the magnet while these coil sets can be accessed and swapped from the opposite side. This ensures that the animal is not disturbed and remains in the same position during the coil change. All PET measurements were performed on a commercial pre-clinical INVEON PET imager (Siemens Healthineers, Erlangen, Germany). An [^18^F]FET-PET tracer was used which was synthesised in-house with a specific radioactivity (< 200 GBq/μmol) [30].

### MR-PET common bed and animal transport system

A 3D printer (Fortus 400mc, Stratasys, MN, USA) using biocompatible polycarbonate was utilised to design and construct the dedicated bed for the rats and the common MR-PET bed adapter. This purpose-built bed contains a freely adjustable bite bar, a nose cone, gas in/out tubes for anaesthesia, a respiratory monitoring pad and a temperature-monitoring unit (SA Instruments, USA). Figure 1b shows the Perspex box (pre-filled with isoflurane) to transport the animal bed along with the anaesthetised animal from the MR unit to the PET site (< 3 min) without disturbance.

### Animal handling and tumour inoculation

All animal measurements were approved by the Animal Protection Committee of the local government (LANUV, North-Rhine-Westphalia, Germany) according to the German Animal Welfare Act and the European Community Council directives regarding the protection of animals used for experimental and scientific purposes (2010/63/EU). All rats weighed between 250 and 310 g and were handled under standard housing conditions (12/12-h light/dark cycle, approximately 22 °C room temperature and 54% humidity, free access to water and food) in the animal facility of Forschungszentrum Juelich. Four Fischer-344 rats purchased from Charles River Laboratories (Germany) were included in this study.

Rodent 9L gliosarcoma cells were implanted into the right striatum of two male rats (rat^1^ and rat^2^), as previously described [31]. 9L cells invade contiguous brain tissue and develop neovascularisation, which is a hallmark of aggressive gliomas, but tumour margins are still well delineated. The other two female rats (rat^3^ and rat^4^) were also used as healthy controls. Tumours were allowed to grow for 11 to 12 days followed by MR and PET measurements. Rats were anaesthetised with 2 to 5% isoflurane in oxygen and placed into the animal scanner, in which they were measured under continuous isoflurane anaesthesia. During both MR and PET measurements, the temperature and respiratory rate of the rats were maintained at around 37.8 °C and between 48 and 55 bpm, respectively. At the time of transportation, isoflurane pre-filled in the box was used to maintain anaesthesia. For the PET scans, a venous catheter was inserted into a tail vein of the rat to deliver the [^18^F]FET tracer.

### Data acquisition scheme and analysis

#### ^1^H MRI, ^23^Na MRI and ^31^P MRS

As described in Fig. 1b, standard adjustments, such as shimming and RF power calibration, were performed using the proton coil prior to multinuclear experiments and the optimised shim values for the brain region were employed for all other nuclei during the MR data acquisition. Experiments were carried out using standard MR sequences cloned from the clinical platform from Siemens since the scanner uses the same hardware apart from coils and magnets. The high resolution structure T_2_-weighted ^1^H images were obtained using a turbo spin-echo (TSE) sequence [32] in all axes.

**Fig. 2:**
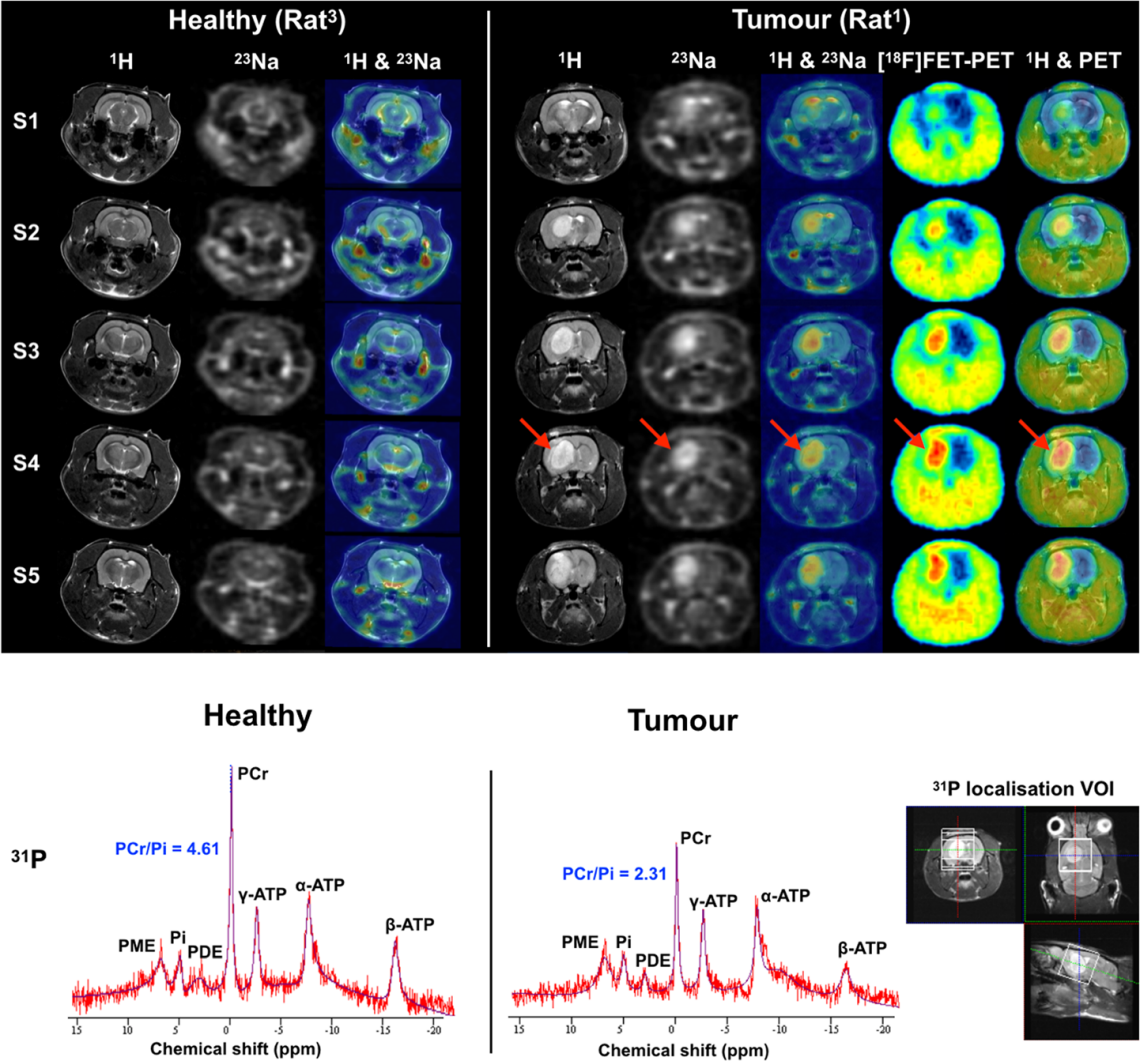
Multinuclear ^1^H MR, ^23^Na MR and [^18^F]FET-PET images (top) and ^31^P spectra with fitting (bottom) of healthy (left, rat^3^) and tumour-bearing (right, pointed out by the red arrow, rat^1^) in vivo rat brain. S1-5, PME, PDE and VOI denote axial slice numbers of the rat brain, phosphomonoester (PME), phosphodiester (PDE) and voxel-of-interest (VOI), respectively

**Fig. 3:**
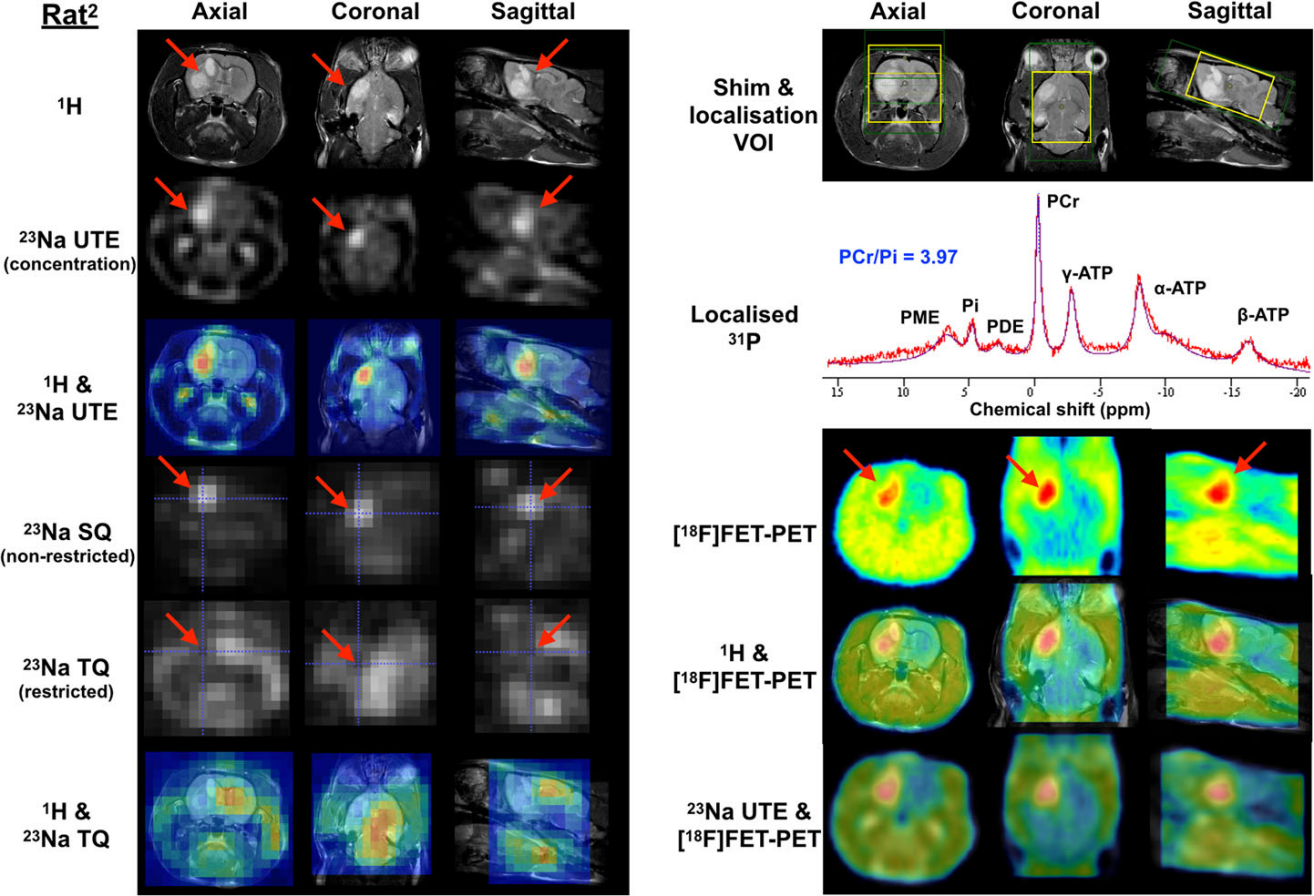
Axial, coronal and sagittal images of ^1^H, ultrashort TE (UTE)/single-quantum (SQ)/triple-quantum-filtered (TQF) ^23^Na (left), co-registered MR (bottom left) and [^18^F]FET-PET images (bottom right) of tumour (pointed out by the red arrow) in vivo rat brain (rat^2^) together with ^31^P MR spectrum with fitting (top right) where the shim-adjusting volume (green box) and ^31^P voxel localisation (yellow box) are shown. ^23^Na images acquired using UTE, SQ and TQ represent total ^23^Na concentration-weighted, non-restricted (mainly extracellular) and restricted (mainly intracellular) ^23^Na, respectively

^23^Na images were collected using either a 3D FLASH [33] for rats^1,3,4^ in Fig. 2 or a simultaneous single-quantum and triple-quantum-filtered MRI of ^23^Na (SISTINA) sequence for a rat^2^ in Fig. 3. Using the advanced SISTINA sequence with multiple phase cycling [10, 34], ultrashort TE (UTE), single-quantum and triple-quantum-filtered ^23^Na images can be generated in a single acquisition, as shown in Fig. 3. This allows for the concurrent acquisition of images leading to the presence of restricted/non-restricted sodium and sodium density-weighted images, which is not possible with the conventional method.

A localised image-selected in vivo spectroscopy (ISIS) sequence [35] was used to acquire ^31^P spectra. ^31^P MR spectra were processed with the use of the advanced method for accurate, robust and efficient spectral fitting (AMARES) provided by jMRUI [36], and the ^31^P metabolites were analysed, such as a PCr/Pi ratio as an example. The detailed MR scan parameters for each experiment are summarised in Table 1. The acquired MR images were then analysed and co-registered using in-house MATLAB (MathWorks, Natick, MA, USA) and ITK-SNAP software [37].

**Table 1:** Detailed MR sequence parameters

	Figure 2			Figure 3		
Nucleus	1.1.1.1.1.1. ^1^H	1.1.1.1.1.2. ^23^Na	1.1.1.1.1.3. ^31^P	^1^H	^23^Na	^31^P
MR sequence	TSE	3D FLASH	ISIS	TSE	SISTINA	ISIS
1.1.1.1.1.4. TR (ms)	3000	40	4000	3000	150	4000
1.1.1.1.1.5. TE (ms)	37	2.85	0.35	37	0.36, 7, 16.2, 25.4, 34.6, 43.8	0.35
1.1.1.1.1.6. No. averages	2	188	360	2	36	520
1.1.1.1.1.7. Resolution (mm^3^)	0.1 **×** 0.1 **×** 1	1 **×** 1 **×** 1	–	0.1 **×** 0.1 **×** 1	2 **×** 2 **×** 2	**–**
1.1.1.1.1.8. VOI (mm^3^)	–	-	11 **×** 11 **×** 11	-	-	11 **×** 15 **×** 18
1.1.1.1.1.9. Scan time (min)	09:51	30:12	24:06	09:51	26:33	34:48
1.1.1.1.1.10. Extra for SISTINA	*BW* = 500/120 Hz/pixel, *Delta* = 40 μs, *Tau* = 7 ms, *RF duration* = 400 μs, 13500 projections					

#### [^18^F]FET-PET

A transmission scan (~ 10 min) with a retractable ^57^Co point source for attenuation and scattering corrections was initially carried out prior to PET data acquisition. The acquisition was then performed in the 3D list mode (~ 65 min) starting with an injection of 40 ± 3 (mean ± range) MBq [^18^F]FET in saline into the tail vein (bolus injection of 0.5 ml in 1 min). Emission data were framed into a dynamic sequence of 6 × 10 s, 5 × 60 s, 5 × 3 min, 10 × 4 min frames. Filtered back-projection with ramp filter (cut-off = 0.5) was employed for the reconstruction of 159 slices, with a voxel size of 0.8 × 0.8 × 0.8 mm^3^. All images were corrected for random coincidences, scatter and attenuation. [^18^F]FET uptakes in the tumour and healthy tissue were expressed as standardised uptake values by dividing the radioactivity (kBq/mL) in the tumour and tissue, respectively, by the radioactivity injected per gram of body weight. Tumour-to-brain ratios (TBRs) were also calculated by dividing the mean value of the tumour by the mean value of normal brain tissue. Summed PET images (18–60 min post-injection) were used for co-registration of MR and PET images. The acquired PET data were analysed using PMOD (PMOD Technologies LLC., Switzerland) and the co-registration with other nuclei was manually carried out with the aid of the landmarking in the body.

## Results

Figure 2 shows a series of five transverse slices of in vivo multinuclear MR (^1^H, ^23^Na and ^31^P) and [^18^F]FET-PET measurements from the brains of healthy rats (left) and the rat^1^ with brain tumours (right). ^1^H MR images present structural details of the rat head and brain and show hyperintensities in the region of the tumour. The anatomical proton images were used as a reference for co-registration of either the ^23^Na or [^18^F]FET-PET images.

The ^23^Na images weighted for total tissue sodium concentration showed a relatively homogenous signal in the healthy brain, while the sodium signal intensity in the tumour region was considerably elevated providing high contrast to the healthy tissue. Ventricles and skin were also clearly distinguishable due to their higher sodium concentration. The tumour exhibits high [^18^F]FET uptake with a similar extent as in the ^1^H MRI. The standardised uptake values (SUV) of the tumour and normal brain tissue were calculated as 1.511 and 0.755, respectively, and the TBR was 2.0. In ^31^P MR spectra, the PCr/Pi ratio is 50% lower than in the normal brain. The voxel-of-interest (VOI) of ^31^P was localised in the tumour region as shown at the bottom right in Fig. 2.

Figure 3 (left) shows ^23^Na images from each slice of axial, coronal and sagittal plane, with different weightings. Using the SISTINA sequence [10], intracellular and extracellular sodium signal contribution can be largely differentiated. UTE and single-quantum (SQ) ^23^Na images show the tissue sodium concentration contrast in the brain with high spatial resolution. It was found that higher sodium concentration in UTE (~ 64%) and in SQ (~ 88%) images was observed in the region of the tumour tissue compared to the normal tissue. This was also in accordance with increased [^18^F]FET uptake (SUVs of tumour/normal = 1.377/0.660 and TBR = 2.1). In contrast, triple-quantum-filtered (TQF) images, also acquired using SISTINA, showed a 32% lower signal in the tumour region. A localised single voxel ^31^P spectrum, also shown in Fig. 3, depicts excellent signal-to-noise ratio (SNR) to clearly distinguish individual ^31^P metabolites.

## Discussion

In this pilot study, we have successfully demonstrated the in vivo feasibility of multinuclear and multimodal rat brain tumour imaging using an ultra-high field MR-PET set-up. The results are based on the previous MR development of a sophisticated RF system which included the RF coils and interface essential for performing multinuclear MR experiments [28]. The development of this system was particularly challenging, as increasing the number of available nuclei in the MR setup is likely to result in substantial SNR loss and degradation of image quality [38–40] if the utmost care is not taken. When comparing the performance of a double- or triple-tuned coil, single-tuned coils are always used as reference coils. Hence, because our proposed coil system comprises only single-tuned coils, which are similar to the reference coils in the literature, it can be assumed that there was no signal loss in any of the measured nuclei. Compared to double- or triple-tuned coils, these single-tuned coils benefit from optimal SNR. However, although there is a temporal penalty associated with switching coils, this is considered to be negligible as the switching can be achieved in under a minute, and animal repositioning is not required during the change of coil [28].

In this study, we have also shown how multinuclear MR in an ultra-high field preclinical scanner can be used in conjunction with [^18^F]FET-PET to verify the potential feasibility of different X-nuclei and the metabolic information thus derived. The results obtained were comparable with previous studies in the literature [10, 31, 41] showing hyperintensity in ^23^Na UTE, high [^18^F]FET uptake in PET images and a reduction of PCr/Pi ratio in the tumour compared to the healthy brain. Changes in ^31^P metabolite ratios, such as PME/PDE, PDE/Pi, PCr/Pi and ATP/Pi, and in intracellular pH appeared to be different in various brain tumour types and grades [41]. Considerable differences in the concentration of ^31^P metabolites were observed in tumour tissue compared with normal, healthy brain tissue. All ^1^H, ^23^Na and [^18^F]FET-PET images appear to specify regions of abnormal contrast. In particular, differences in ^23^Na imaging were comparable to the results of a previous study [42], in which information obtained with ^23^Na imaging was used to aid prediction of the mutational status of the enzyme isocitrate dehydrogenase (IDH). The molecular genetic profile of cerebral gliomas such as IDH mutation is of increasing importance for the individual treatment strategy of patients [43]. Since this requires a biopsy of the brain tumour, there is a major interest in obtaining such information non-invasively using modern molecular imaging techniques. In the study mentioned above, IDH-mutated gliomas exhibited a significant increase in total ^23^Na concentration, a relative increase of ^23^Na ions with unrestricted mobility and a relative decrease of ^23^Na ions with restricted mobility. In contrast, none of the IDH wild-type gliomas showed this pattern, and all sodium imaging parameters were significantly different compared with those of IDH-mutated gliomas [42]. It is anticipated that due to the availability of corresponding glioma models with and without IDH mutation, in the future, it will be possible to analyse such findings with multinuclear and multimodal in vivo rat brain tumour imaging [44]. These investigations are of great importance since the exact mechanisms influencing the sodium signal may be caused by various factors. Thus, a relative decrease in the concentration of sodium ions with restricted mobility may be influenced by an increase in the intracellular or extracellular space. The elucidation of this requires a detailed analysis of histological parameters, which are readily available in animal models.

The combination of multinuclear MR (more than three nuclei) and PET in, for example, brain tumour models with specific genetic mutations will enable exploration of the physiological background of signal alterations and the identification of the optimal combination of imaging parameters for the non-invasive characterisation of the molecular profile of tumours. Depending on the MR nuclei and the PET tracer of interest, the acquisition scheme can be flexibly redesigned, for example, to validate cerebral oxygen consumption mapping [45], to investigate the cellular regulatory activity using the Na^+^/K^-^ pump [46] or to monitor pyruvate and lactate in the tumour [47].

Interest in simultaneously operating MR-PET hybrid clinical systems is increasing, due to the reduction in acquisition time, which can be reallocated for adding other nuclei in the acquisition process and to study multiple biological parameters simultaneously and with high spatial and high temporal resolution [4, 48–50]. Notwithstanding the fact that our study was performed sequentially on two separate scanners, it shows the potential for the future development of ultra-high field, multinuclear MR-PET hybrid scanners capable of simultaneous operation.

## Conclusions

The combination of multinuclear MR and PET offers complementary information for the investigation of brain tumours. The unique integration of MR-PET, as proposed, allows in vivo preclinical multinuclear MR-PET experiments to be conducted without compromising any aspects of multinuclear MR or PET qualities. Since using conventional MRI does not always provide the required level of detail to determine the grade of brain tumours, to differentiate radiation necrosis from tumour recurrence, or to detect treatment responses, X-nuclei MR in combination with PET may be a more appropriate strategy to fulfil this purpose.

## Data Availability

The datasets generated during and/or analysed during the current study are available from the corresponding author on reasonable request.

## Abbreviations

[^18^F]FET: O-(2-[^18^F]-fluoroethyl)-L-tyrosine
^23^Na: Sodium-23
^31^P: Phosphorus-31
AMARES: Advance method for accurate, robust and efficient spectral fitting
ATP: Adenosine triphosphate
IDH: Isocitrate dehydrogenase
ISIS: Localised image-selected in vivo spectroscopy
MRI: Magnetic resonance imaging
MRS: Magnetic resonance spectroscopy
PCr: Phosphocreatine
PDE: Phosphodiester
PET: Positron emission tomography
Pi: Inorganic phosphate
PME: Phosphomonoester
SISTINA: Simultaneous single-quantum and triple-quantum filtered MRI of ^23^Na
SNR: Signal-to-noise ratio
SQ: Single-quantum
SUV: Standardised uptake values
TBR: Tumour-to-brain ratio
TQF: Triple-quantum-filtered
T/R: Transmit/receive
TSE: Turbo spin echo
UTE: Ultrashort TE
VOI: Voxel-of-interest
